# Validation of the Canadian Version of the Shame and Stigma Scale for Head and Neck Cancer Patients

**DOI:** 10.3390/curroncol30080548

**Published:** 2023-08-11

**Authors:** Irene Bobevski, David W. Kissane, Justin Desroches, Avina De Simone, Melissa Henry

**Affiliations:** 1 School of Medicine, University of Notre Dame Australia, Darlinghurst, NSW 2010, Australia; david.kissane@nd.edu.au; 2 Department of Psychiatry, School of Clinical Sciences, Monash Health and Monash University, Clayton, VIC 3168, Australia; 3 Faculty of Medicine, McGill University, Montreal, QC H3A 0G4, Canada; justin.desroches@mail.mcgill.ca (J.D.);; 4 Gerald Bronfman Department of Oncology, McGill University, Montreal, QC H3A 0G4, Canada; 5 Lady-Davis Institute for Medical Research, Jewish General Hospital, Montreal, QC H3T 1E2, Canada

**Keywords:** shame, stigma, head and neck cancer, psychometrics, quality of life, disfigurement, appearance, speech, regret, body image

## Abstract

Cancers of the head and neck and their treatment can cause disfigurement and loss of functioning, with a profound negative impact on the person’s self-image and psychosocial wellbeing. This can lead to experiences of shame and stigma, which are important targets for psychosocial interventions. Accurate measurement and identification of these problems enables clinicians to offer appropriate interventions and monitor patients’ progress. This study aimed to validate the Canadian version of the Shame and Stigma Scale (SSS) among French- and English-speaking head and neck cancer patients. Data from 254 patients from two major Canadian hospitals were analysed. The existing four-factor structure of the SSS was supported, with the following subscales: Shame with Appearance, Sense of Stigma, Regret, and Social/Speech Concerns. The Canadian SSS showed adequate convergent and divergent validity and test–retest reliability. Rasch analysis suggested scale improvement by removing two misfitting items and two items with differential functioning between French- and English-speaking patients. The final 16-item scale version was an adequate fit with the Rasch model. The SSS provides more accurate measures for people with high levels of shame and stigma, and thus has utility in identifying patients with more severe symptoms who may be in need of psychosocial interventions.

## 1. Introduction

Cancers of the head and neck and their treatment can cause various degrees of disfigurement and loss of functioning, with a profound negative impact on the person’s self-image and psychological and social wellbeing [1,2,3,4]. This can lead to experiences of shame and stigma [5]. Shame is a psychological state in which the person experiences a sense of disgrace, dishonour or humiliation, and a desire to cover up, hide, or escape [6]. Stigma involves social disapproval regarding the person’s identity or disfigurement [7]. Experiences of shame and stigma in cancer patients are associated with low self-esteem, depression, social isolation [1,8,9,10], and deteriorating family relationships despite physical improvement [11]. Head and neck cancer patients also often experience guilt and regret about past behaviours [12], such as smoking or drinking, that may have contributed to the cancer [13,14].

Shame, stigma, and regret are important targets for psychosocial interventions with head and neck cancer patients in order to help them modify their negative perceptions and develop acceptance and accommodation [15]. Accurate measurement [16] and identification of these problems enables clinicians to offer appropriate interventions and monitor patients’ progress. It also contributes to a better understanding of the prevalence of shame and stigma and their impact on patients’ lives [15].

In response to the above needs, Kissane et al. [15] developed and validated the Shame and Stigma Scale (SSS) at the Memorial Sloan-Kettering Cancer Center in the US. Since then, the scale has been translated and validated in seven different countries [17] and has been found to be a reliable and valid measure of shame and stigma.

The aim of the current study was to validate the scale among French- and English-speaking Canadian head and neck cancer patients. It is important to explore the scale’s functioning in different cultural and language contexts, and to adapt it to these specific needs if necessary. This appears particularly important considering the known detrimental effects of shame and stigma on physical and mental health outcomes and its target as a public health concern by Canada’s Chief Public Health Officer [18].

## 2. Materials and Methods

### 2.1. Study Population and Procedure

Patients were recruited through the Otolaryngology–Head and Neck Surgery departments of two large Canadian hospitals affiliated with McGill University. Eligible patients had undergone a disfiguring surgery less than three years from the time of referral. The type of surgery was defined in consultation with medical teams. Patients were mailed the study questionnaires, which they returned by mail in prestamped, preaddressed envelopes. Two weeks later, patients were resent the questionnaires again for test–retest validation. Completion of the questionnaire required 20–25 min. Patients were able to complete the questionnaires at a time convenient to them to minimise burden and reduce fatigue. The recruitment procedure is described in more detail elsewhere, in a study led by one of the authors, Melissa Henry [19].

### 2.2. Measures

The following well-validated measures were used in the current study: Shame and Stigma Scale (SSS) [15] (the English version and a French translation were used), Body Image Coping Strategies Inventory (BICSI) [20], Body Image Scale (BIS) [21], Hospital Anxiety and Depression Scale (HADS) [22,23], Centre of Epidemiologic Studies Depression (CES-D) [24], Illness Intrusion Ratings Scale (IIRS) [25,26], Functional Assessment of Cancer Therapy—General (FACT-G) [27], and Functional Assessment of Cancer Therapy—Head and Neck (FACT-H&N) [28], Buss–Perry Aggression Questionnaire—Short Form (BPAQ) [29,30], and Marlowe–Crown Social Desirability—Short Form (MCSD) [31,32]. The SSS was translated into French using backtranslation [33].

### 2.3. Statistical Analysis

To assess the factor structure of the SSS, exploratory factor analysis was conducted. The factor analysis used the matrix of polychoric correlations of the items rather than the Pearson correlations. This is more appropriate for ordinal data [34]. An oblique promax rotation was used, allowing for correlations between the factors. Eigen values ≥1 and the scree plot were considered in factor selection, as well as conceptual considerations of factor interpretability.

Descriptive statistics, such as means, medians, and response frequencies, were derived for each SSS subscale and for each item. Internal consistency reliabilities (Cronbach’s α) and test–retest reliabilities were also calculated. Spearman’s rho was used for the test–retest reliability due to the skewness of the data.

Convergent and divergent validity were examined using calculation of Spearman’s rho correlations between the SSS and its subscales and the other measures used in the study. It was expected that the SSS would have stronger associations with appearance-related scales, such as the BICSI, BIS, FACT-G, and FACT H&N, as well as at least moderate correlations with psychological distress (the HADS and CES-D) and interference of illness with life (IIRS). The SSS was expected to have relatively weaker associations with aggression (PAQ) and social desirability (MCSD) scales.

Rasch [35] analysis was used to further investigate the psychometric properties of the SSS based on item-level information and the respondents’ pattern of responses to each item. Rasch is a probabilistic model that estimates each respondent’s ability (or level of the measured attribute) and each item’s difficulty (or level of the attribute measured by the item) on the same scale with logits as the measuring units [36]. Rasch analysis was used to evaluate overall model fit, item fit, unidimensionality, potential item bias between subgroups of respondents, and how well the items targeted respondents. A good overall model fit is indicated by a nonsignificant λ^2^ (with a Bonferroni adjustment for the number of items), and an overall item fit residual standard deviation below the accepted limit of <1.5. Individual item fit is indicated by item-standardised residuals within the accepted range of >−2.5 and <2.5. High item interdependencies are indicated using item residual correlations ≥0.2 [37]. Internal consistency was assessed with the Person Separation Index (PSI) estimated using Rasch, and with Cronbach’s α. The PSI is interpreted similarly to α, with values ≥0.70 considered good [37]. Targeting (or the alignment of the item difficulty and person ability levels) was assessed by examining the person–item threshold distribution. Differential item functioning (DIF) refers to item bias or differential item performance [38]. DIF for the language in which the scale was completed (French or English), sex, age, and education were examined with analysis of variance, comparing Rasch scores for each level of these variables across each item. Item thresholds (the points between each adjacent response category where either of the adjacent responses is equally likely) were tested for disordering (when responses are not ordered as expected, low to high). The unidimensionality of each subscale and the whole scale was assessed by using *t*-tests between the Rasch-derived scores of subsets of items identified using principal component analysis on the residual correlations. Unidimensionality is confirmed if significantly less than 5% of the *t*-tests are significant [39]. A minimum of 150 participants is required to estimate item difficulty within ±0.5 logits accuracy [40].

Rasch analysis was conducted with RUMM2030 [41] software using the partial credit model [42]. All other analyses were conducted with Stata 17 [43].

## 3. Results

### 3.1. Description of the Study Population

Data from 254 patients were collected and analysed. Of the study population, 55% were recruited from the Jewish General Hospital and 44% from the McGill University Health Centre. The questionnaires were completed in French by 53% and in English by 47% of the sample. Of the sample, 67% were male, median age was 67 years with a range of 23–98 years, 64% were partnered, 34% had completed at least high school, and 42% had tertiary education. The most common forms of cancer were cutaneous (38%), oral cavity (21%), and oropharynx (12%). Of the sample, 14% had advanced stage IV cancer, 29% stage III, 23% stage II, and 18% stage I. The demographics of the study population are described in detail elsewhere [19].

### 3.2. Factor Analysis of the Shame and Stigma Scale

Four factors were derived from the exploratory factor analysis (Table 1), consistent with Kissane et al.’s [15] original validation of the scale. There were several cross-loading items that had a lower or similar loading on two factors, but they were retained in the factor they conceptually fitted most with, in line with the original scale. Item 9 was the only one that did not load as expected. Overall, the results supported the four-factor structure of the original scale. The four factors were: Sense of Stigma (eight items), Shame with Appearance (six items), Regret (three items), and Social/Speech Concerns (three items). The correlations between the factors were moderate in size and similar to those obtained in Kissane et al.’s [15] original validation, ranging from 0.27 to 0.46.

### 3.3. Descriptive Statistics for the Subscales and Items of the SSS

Table 2 shows that the first three scales had good internal consistency (Cronbach’s α from 0.76 to 0.82) and test–retest reliability (Spearman’s rho from 0.60 to 0.73). The Social/Speech Concerns subscale had lower internal consistency of 0.52. The Sense of Stigma Scale had a substantial floor effect, with nearly half the scores being zero. The medians and means of the subscales and the complete scales were fairly low.

Table 3 shows good corrected correlations between the items and their subscales and the total scale. Item 20 has the lowest correlation with the total scale (0.24). For most items, very low proportions of the participants obtained high scores above 3. There was a substantial floor effect for many of the items. The results in Table 2 and Table 3 indicate skewed data, with only a small proportion of participants scoring in the high range of the item response categories.

The convergent and divergent validity correlations (Table 4) follow the expected pattern. The Shame with Appearance scale had the strongest correlations with the appearance-related scales (BICSI, BIS, FACT-G, and FACT H&N). All SSS subscales were weakly to moderately correlated to the depression and anxiety scales (GADS and CES-D) and illness interference with life (IIRS). The weakest correlations for all subscales were with the aggression (PAQ) and social desirability (MSCD) scales.

### 3.4. Rasch Analysis

A summary of the Rasch analysis for each SSS subscale and the total scale is presented in Table 5. The Rasch analysis suggested that the Canadian version of the SSS would be improved by removing four items: 8, 9, 17, and 20.

On the Shame with Appearance subscale, item 8 (“I am distressed by the changes in my face and neck”) showed significant DIF in language, with respondents who completed the scale in English consistently scoring higher than expected on this item and those who completed it in French scoring lower. After item 8 was removed, there was no remaining DIF, and the model fit and PSI were adequate.

The Sense of Stigma subscale initially showed some misfit, with a significant χ^2^ for the overall model fit, although the item residual standard deviation and the PSI were within acceptable limits. This subscale contained item 9 (“I feel others consider me responsible for my cancer”), which upon factor analysis loaded on only the Regret subscale. In the original validation of the SSS, item 9 was included in the Sense of Stigma subscale for conceptual reasons, but upon factor analysis it also loaded on Regret and Social/Speech Concerns. In the Canadian version, removing item 9 improved the fit of the Sense of Stigma subscale, with the overall model fit χ^2^ becoming nonsignificant, and the item residual standard deviation and the PSI improving slightly.

Item 17 (“I feel sorry about the things I have done in the past”) of the Regret subscale showed significant DIF for language, with French speakers scoring higher than expected and English speakers lower. Removing Item 17 reduced the PSI from 0.56 to 0.37; however, α remained adequate at 0.71. Since this is only a two-item scale with items targeted towards high-scoring respondents, this model fit is acceptable despite the low PSI.

The Social/Speech Concerns subscale showed an overall model misfit with a highly significant χ^2^, and the PSI could not be estimated properly. Cronbach’s α was also low (0.52). All items showed misfit with a highly significant χ^2^ (*p* < 0.0001), with item 20 (“I am able to join conversations”) having a large fit residual (2.35), close to the ≤2.5 limit. After deleting item 20, an adequate model fit was obtained and α improved to 0.79, suggesting good internal consistency. Item 20 also showed misfit in Kissane et al.’s [15] original validation of the SSS, with an indication that some patients may have responded to it as a negatively worded item. Item 20 is a positively worded item after a row of 12 negatively worded items, which may have caused confusion among respondents.

**Figure 1:**
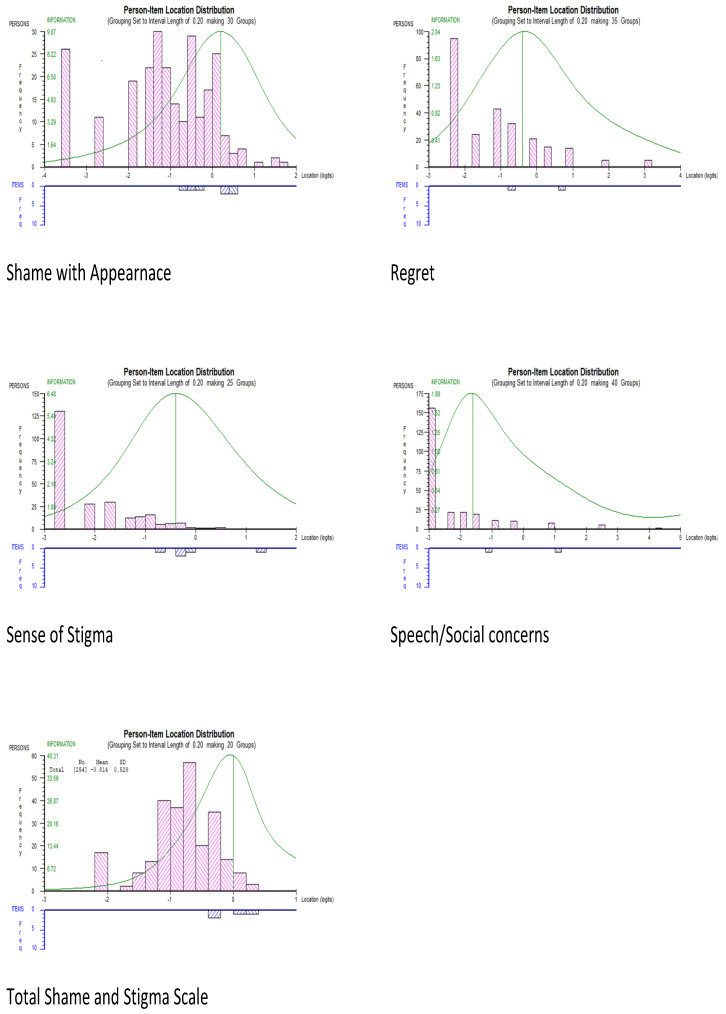
Item-person location maps for the SSS subscales and the total scale. Note: the locations in logits are shown for persons at the top and for items at the bottom of the *X*-axis.

The PSI for the Sense of Stigma, Regret, and Social/Speech Concerns scales were below the acceptable value of 0.70, while Shame with Appearance and the total scale had acceptable PSIs. All subscales and the total scale had acceptable Cronbach’s α values. Both the PSI and α are measures of internal consistency reliability. While α is calculated from raw scores, the PSI is based on estimated Rasch person locations. If the scale’s item difficulties and person abilities are well aligned, α and PSI are close in value. A misalignment in a skewed distribution results in a discrepancy between α and PSI, since with more extreme person scores, the error variance in the PSI increases but α remains more constant [41]. In the current sample, there was a large proportion of respondents who mainly endorsed zero or the low end of the item categories, few high scoring respondents, and subscales with a small number of items. This would explain the discrepancy between PSI and α, as well as obtaining low PSIs for the most misaligned subscales. In the total SSS, with an increased number of items covering a wider range of person abilities, the PSI improved. Overall, the acceptable α values, combined with good fit of the models, indicate that the subscales had adequate psychometric properties. However, they are better targeted at a population with a higher level of shame and stigma than the current sample. Figure 1, showing the item–person location distributions, illustrates that most items were targeted towards the higher range of difficulties, while persons are mainly located at the lower end of the distribution.

All subscales and the total scale were unidimensional, with none having significantly more than 5% of significant unidimensionality *t*-tests.

All item thresholds, except for item 5 (“I feel people stare at me”), were disordered. Collapsing the item response options into fewer categories (Never, Seldom/Sometimes, Often/All the time) resulted in ordered thresholds, but did not affect the fit of the models. Collapsing of categories results in loss of statistical information and decreases reliability. Disordered thresholds may not always represent a violation of the intended order of item response categories. They may be a result of low frequencies of responses in some categories [42,44,45,46]. In our sample, there was a high proportion of zero (“Never”) responses and low proportions for most items of “Often” or “All the time” responses. In Kissane et al.’s [15] original validation of the SSS, in which the data were less skewed, the thresholds were ordered. Kissane et al.’s sample was restricted to oral cavity cancers with potentially more severe symptoms, whereas in our sample, 38% of patients had cutaneous cancers with less severe symptoms, thus restricting the SSS range. Furthermore, we found that in our data, the average Rasch estimated person abilities increased monotonically as item response categories advanced from 0 to 4 for each SSS item. This suggests that patients responded to the items as intended and the item categories represent advancing levels of shame and stigma [45,46]. Based on the above evidence, it was decided to retain the existing item categories.

The final version of the Canadian SSS is presented in Appendix A.

## 4. Discussion

Despite the morbidity that the diagnosis and treatment of head and neck cancer brings, there has been a relative paucity of validated measures that are specific to the needs of this population [47]. Here, we take just such a measure and validate it for the mixed language needs of a Canadian population. The only other related measure that has been validated in English and French in a Canadian context is the McGill Body Image Concerns Scale for use in head and neck oncology (MBIS-HNC). The latter comprises two subscales: social discomfort and negative self-image. It focuses more specifically on changes in appearance, function, and senses as a result of head and neck cancer treatments [19]. Rodriguez et al. [19] reported moderate to high correlations between the MBIS-HNC and the SSS subscales (0.49 with Regret, 0.50 with Social/Speech Concerns, 0.55 with Sense of Stigma, and 0.72 with Shame about Appearance). This suggests that the two scales have good convergent validity but measure different constructs.

We have shown the Shame and Stigma Scale to be a reliable and valid measure and to have adequate psychometric properties in a Canadian population of cancer patients. The factor structure of the original SSS was supported, with the existing four subscales: Shame with Appearance, Sense of Stigma, Regret, and Social/Speech Concerns. The Canadian version of the SSS showed adequate convergent and divergent validity and test–retest reliability. The SSS followed the expected pattern, of having stronger associations with measures of concerns about appearance, psychological distress, and life interference from illness. The SSS had weaker associations with less related constructs, such as aggression and social desirability.

We suggest scale improvements by removing two misfitting items (items 9 and 20) and two items with differential functioning between French- and English-speaking patients (items 8 and 17) to adapt it to the Canadian population. Item 9 did not load as expected in the factor analysis and caused model misfit in the Rasch analysis. This item also cross-loaded on multiple factors in Kissane et al.’s [15] validation. Item 20 caused misfit in the Rasch model and was also found problematic in Kissane et al.’s validation. It was important to remove the differentially functioning items 8 and 17 so that the scale functioned equivalently for both French- and English-speaking Canadians. The final scale version fit adequately with the Rasch model. The unidimensionality of the subscales and total scale was confirmed.

In comparison to our study, validations in five other countries also supported the four-factor structure of the SSS. The Taiwanese [48] and Malay [49] versions extracted five and three factors, respectively. In the Malay [49] version, all four positively worded items (1, 4, 7, and 20) were removed, and in the Chinese [50] version, items 1 and 4 were removed, since they did not perform well. These two studies raised issues about translating oppositely worded items to different cultures. We note that the positively worded item 20 also exhibited problematic fit in our study and in the original validation [15].

Our study, as well as Kissane et al.’s [15] original validation, showed that the items of the SSS are targeted towards higher levels of shame and stigma severity. This means that the scale provides more accurate measures for people with high levels of shame and stigma. Thus, the SSS has utility in identifying patients with severe levels of shame and stigma who may be in need of psychosocial intervention. The SSS could also be useful for monitoring the progress of interventions, such as counselling or psychosocial therapies.

### Strengths and Limitations

The study had a sample of adequate size and representativeness. Our sample size of 254 enabled a high accuracy of Rasch estimation of item difficulty, within at least ±0.5 logits. Patients from two different health settings and a range of socioeconomic groups were recruited, and French- and English-speaking participants were approximately equally represented. A limitation of this study is that most patients had low levels of shame and stigma, while the SSS is targeted towards the high end of the distributions. The scale would provide less precise assessment for people with low levels of shame and stigma. For many of the SSS items the highest categories “Often” and “Sometimes” had very low response frequencies. This may have contributed to disordered thresholds in the Rasch model [42,44,45,46]. Our data indicated that, nevertheless, the response options functioned as intended. The Rasch estimated person abilities (levels of shame and stigma) increased monotonically with higher response categories for all items [45,46]. However, further investigation of how the item response categories and Rasch estimated thresholds function with less skewed data is warranted in future studies. Further work to investigate how the SSS generalises to other types of cancer and other cultural groups is also needed.

## 5. Conclusions

This study provided evidence of the reliability and validity of the SSS in a Canadian population of cancer patients. The SSS is a promising tool for identifying patients with high levels of shame and stigma in clinical settings in Canada so that appropriate psychosocial interventions can be offered. In future research with the SSS, it would be important to investigate differences in experiences of head and neck cancers between men and women, as well as various sociodemographic and cultural groups.

## Figures and Tables

**Table 1:** Exploratory factor analysis using correlations for ordinal data (polychoric correlations) with promax rotation (allowing correlations between the factors).

Items	Factor 1 Sense of Stigma	Factor 2 Shame with Appearance	Factor 3 Regret	Factor 4 Social/Speech Concerns
1. I like my appearance	0.63	**0.71**		
2. I avoid looking at myself in the mirror	0.45	**0.41**		
3. I am ashamed of my appearance		**0.49**		
4. I am happy with how my face or neck looks		**0.74**		
5. I feel people stare at me		**0.52**		
6. I avoid meeting people because of my looks	0.51	**0.50**		
7. I enjoy going out in public		**0.57**		
8. I am distressed by the changes in my face or neck		**0.57**		
9. I feel others consider me responsible for my cancer			0.44	
10. I am embarrassed when I tell people my diagnosis	**0.68**			
11. I feel ashamed for having developed cancer	**0.55**			
12. People avoid me because of my cancer	**0.41**			0.49
13. I have an urge to keep my cancer a secret	**0.74**			
14. I sense that others feel strained when around me	**0.35**			0.35
15. I have a strong feeling of regret			**0.64**	
16. I would do many things differently if given a second chance			**0.78**	
17. I feel sorry about things I have done in the past			**0.75**	
18. I am embarrassed by the change in my voice				**0.77**
19. I avoid talking with others				**0.66**
20. I am able to join conversations				**0.42**

Note: Factor loadings ≥0.35 are shown. Bold print indicates the subscale upon which items are scored.

**Table 2:** Descriptive statistics and internal consistency for the Shame and Stigma subscales and for the total score.

	No. of Items	% with 0 Scores	Max. Possible Score	Median (IQR)	Mean (SD)	Cronbach’s α	Test–Retest (Spearman’s Rho)
Shame with Appearance	8	10.5%	32	6.0 (3.0–11.0)	7.5 (6.2)	0.82	0.68 *
Sense of Stigma	6	47.2%	24	1.0 (0.0–4.0)	2.3 (3.4)	0.76	0.73 *
Regret	3	28.0%	12	3.0 (0.0–5.0)	3.2 (3.0)	0.79	0.60 *
Social/Speech Concerns	3	35.1%	12	2.0 (0.0–4.0)	2.6 (2.6)	0.52	0.57 *
Total Scale	20	2.9%	80	12.0 (6.0–22.0)	15.4 (11.9)	0.88	0.72 *

IQR = interquartile range; SD = standard deviation; * *p* < 0.0001.

**Table 3:** Percentage of low and high item scores and corrected item correlations with the total scale and the subscales.

Item	% with 0 Scores	% Scoring ≥ 3	Corrected Correlation with Total Scale	Corrected Correlation with Subscale
Shame with Appearance				
1. I like my appearance	25.5	17.6	0.61	0.71
2. I avoid looking at myself in the mirror	63.9	9.8	0.56	0.65
3. I am ashamed of my appearance	69.0	5.1	0.60	0.66
4. I am happy with how my face or neck looks	26.1	29.3	0.56	0.69
5. I feel people stare at me	56.3	11.4	0.59	0.65
6. I avoid meeting people because of my looks	75.0	3.1	0.66	0.69
7. I enjoy going out in public	41.0	19.9	0.56	0.64
8. I am distressed by the changes in my face or neck	64.1	7.0	0.62	0.71
Sense of Stigma				
9. I feel others consider me responsible for my cancer	79.7	4.3	0.49	0.57
10. I am embarrassed when I tell people my diagnosis	76.2	3.9	0.61	0.81
11. I feel ashamed for having developed cancer	79.4	4.0	0.59	0.75
12. People avoid me because of my cancer	86.5	3.6	0.52	0.59
13. I have an urge to keep my cancer a secret	74.4	6.7	0.46	0.67
14. I sense that others feel strained when around me	71.6	3.2	0.63	0.69
Regret				
15. I have a strong feeling of regret	62.6	8.7	0.68	0.81
16. I would do many things differently if given a second chance	40.9	22.5	0.57	0.88
17. I feel sorry about things I have done in the past	40.6	12.6	0.55	0.83
Social/Speech Concerns				
18. I am embarrassed by the change in my voice	69.8	12.3	0.57	0.77
19. I avoid talking with others	70.6	4.0	0.71	0.74
20. I am able to join conversations	43.9	21.7	0.24	0.69

**Table 4:** Convergent and divergent validity: Spearman’s rho correlations.

	BICSI Appearance Fixing	BICSI Avoidance	BIS	HADS Depression	HADS Anxiety	CES-D	IIRS	FACT-G	FACT H&N	BPAQ	MCSD
Shame with Appearance	0.54	0.51	0.63	0.54	0.52	0.52	0.45	−0.65	−0.52	0.28	−0.32
Sense of Stigma	0.48	0.48	0.50	0.41	0.48	0.43	0.45	−0.42	−0.42	0.36	−0.33
Regret	0.43	0.36	0.42	0.43	0.41	0.37	0.41	−0.40	−0.41	0.32	−0.32
Social/Speech Concerns	0.23	0.26	0.37	0.39	0.32	0.39	0.30	−0.50	−0.50	0.18	−0.15
Total Scale	0.58	0.55	0.66	0.57	0.60	0.58	0.52	−0.69	−0.61	0.35	−0.36

Note: All correlations are significant with *p* < 0.0001.

**Table 5:** Rasch analysis summary.

Analysis	Item Residual Mean (SD)	Person Residual Mean (SD)	χ^2^	*p*	PSI	α	Unidimensionality % Significant *t*-Tests (95% CI)
Shame with Appearance (8 items) DIF for item 5 and item 8 for language.	0.80 (1.01)	−1.14 (1.15)	21.37	0.165	0.73	0.82	7.09 (4.41–9.76)
Final (7 items): Item 8 removed due to DIF. No remining DIF for any items after item 8 removed.	0.98 (0.62)	−0.37 (1.10)	28.08	0.014	0.72	0.79	7.48 (4.79–10.16)
Sense of Stigma (6 items)	−0.29(0.98)	−1.88 (0.80)	19.85	0.003 *	0.21	0.76	0
Final (5 items): Item 9 not included due to model misfit and not loading on appropriate factor.	−0.14 (0.74)	−0.32 (1.08)	12.41	0.03	0.22	0.77	0
Regret (3 items)	0.35 (0.76)	−0.40 (0.97)	4.36	0.225	0.56	0.79	0.79 (−1.89–3.46)
Final (2 items): Item 17 deleted due to DIF	0.36(0.55)	−0.50 (1.06)	5.03	0.081	0.37	0.71	0
Social/Speech Concerns (3 items) Misfit in item 20.	0.73 (1.44)	−0.11 (0.90)	93.24	0.000 *	−0.16	0.52	0.39 (−2.29–3.07)
Final (2 items): Item 20 removed	0.63 (1.04)	−0.29 (0.82)	4.92	0.085	0.48	0.79	0.39 (−2.29–3.07)
Total Scale (16 items) (With four subscales and items 8, 9, 17, 20 deleted)	−0.47 (1.27)	−0.32 (0.82)	25.55	0.012	0.62	0.70	2.36 (−0.318–5.04)

SD = standard deviation; CI = confidence interval. * Significant *p* at the 0.05 level after a Bonferroni adjustment for the number of items.

## Data Availability

Data not publicly available due to privacy and ethical restrictions.

## Appendix A

**Table A1:** Canadian version of the Shame and Stigma Scale in Head and Neck Cancer.

	Never	Seldom	Sometimes	Often	All the Time
1. I like my appearance	0	1	2	3	4
2. I avoid looking at myself in the mirror	0	1	2	3	4
3. I am ashamed of my appearance	0	1	2	3	4
4. I am happy with how my face or neck looks	0	1	2	3	4
5. I feel people stare at me	0	1	2	3	4
6. I avoid meeting people because of my looks	0	1	2	3	4
7. I enjoy going out in public	0	1	2	3	4
8. I am embarrassed when I tell people my diagnosis	0	1	2	3	4
9. I feel ashamed for having developed cancer	0	1	2	3	4
10. People avoid me because of my cancer	0	1	2	3	4
11. I have an urge to keep my cancer a secret	0	1	2	3	4
12. I sense that others feel strained when around me	0	1	2	3	4
13. I have a strong feeling of regret	0	1	2	3	4
14. I would do many things differently if given a second chance	0	1	2	3	4
15. I am embarrassed by the change in my voice	0	1	2	3	4
16. I avoid talking with others	0	1	2	3	4

Subscales. Shame with Appearance: items 1–7. Sense of Stigma: items 8–12. Regret: items 13–14. Social/Speech concerns: items 15–16.
